# A national perspective on prenatal testing for mitochondrial disease

**DOI:** 10.1038/ejhg.2014.35

**Published:** 2014-03-19

**Authors:** Victoria Nesbitt, Charlotte L Alston, Emma L Blakely, Carl Fratter, Catherine L Feeney, Joanna Poulton, Garry K Brown, Doug M Turnbull, Robert W Taylor, Robert McFarland

**Affiliations:** 1 Wellcome Trust Centre for Mitochondrial Research, The Medical School, Institute for Ageing and Health, Newcastle University, Newcastle-upon-Tyne, UK; 2 NHS Specialised Services for Rare Mitochondrial Disorders of Adults and Children UK, Oxford, UK; 3 Oxford Medical Genetics Laboratories, Oxford University Hospitals NHS Trust, Oxford, UK; 4 Nuffield Department of Obstetrics and Gynaecology, University of Oxford, John Radcliffe Hospital, Oxford, UK; 5 Department of Biochemistry, University of Oxford, Oxford, UK

## Abstract

Mitochondrial diseases affect >1 in 7500 live births and may be due to mutations in either mitochondrial DNA (mtDNA) or nuclear DNA (nDNA). Genetic counselling for families with mitochondrial diseases, especially those due to mtDNA mutations, provides unique and difficult challenges particularly in relation to disease transmission and prevention. We have experienced an increasing demand for prenatal diagnostic testing from families affected by mitochondrial disease since we first offered this service in 2007. We review the diagnostic records of the 62 prenatal samples (17 mtDNA and 45 nDNA) analysed since 2007, the reasons for testing, mutation investigated and the clinical outcome. Our findings indicate that prenatal testing for mitochondrial disease is reliable and informative for the nuclear and selected mtDNA mutations we have tested. Where available, the results of mtDNA heteroplasmy analyses from other family members are helpful in interpreting the prenatal mtDNA test result. This is particularly important when the mutation is rare or the mtDNA heteroplasmy is observed at intermediate levels. At least 11 cases of mitochondrial disease were prevented following prenatal testing, 3 of which were mtDNA disease. On the basis of our results, we believe that prenatal testing for mitochondrial disease is an important option for couples where appropriate genetic analyses and pre/post-test counselling can be provided.

## Background

Mitochondrial diseases are clinically and genetically heterogeneous conditions affecting >1 in 7500 live births^1^ and causing significant morbidity and mortality.^2, 3^ Genetic counselling for families with mitochondrial disease can provide some unique and difficult challenges, particularly in relation to disease transmission and prevention. A clear understanding of the inheritance patterns in families with mitochondrial disease, the reproductive and prenatal testing options available, their application to mitochondrial disease, and the risks involved is crucial if accurate and appropriate advice is to be imparted to prospective parents.

Mitochondria are small multifunctional intracellular organelles present in all eukaryotic cells and primarily responsible for generating adenosine triphosphate by oxidative phosphorylation. Their efficient functioning is determined by two genomes; the nuclear genome and also the maternally inherited 16.6-kb mitochondrial genome.^4, 5^ Disease may occur as a result of mutations in either of these genomes. Defects in nuclear DNA (nDNA) can cause problems with mitochondrial DNA (mtDNA) maintenance and repair, defects in translation or structural defects of respiratory chain complexes.^5, 6^ mtDNA is present in multiple copies within cells and in oocytes the copy number can exceed 1 × 10^5^ per cell.^7^ A consequence of this high intracellular copy number is the phenomenon of heteroplasmy, where two or more species of mtDNA co-exist. This situation arises when a mtDNA mutation occurs within only a proportion of the mtDNA present. Importantly though, disease will only occur when a tissue-specific threshold has been exceeded (usually >60%). This threshold is not the same for all mtDNA mutations and the level of heteroplasmy may change slowly with time.^4, 5, 8, 9^

Patients may develop their first symptoms in adulthood and it is not unusual for the mtDNA mutation to be transmitted to children before their mother becomes symptomatic. In addition, asymptomatic mothers with low levels of mtDNA mutation may have symptomatic children with very high levels because of a genetic phenomenon known as the ‘mtDNA genetic bottleneck'. A main component of this bottleneck occurs during formation and initial divisions of primordial germ cells when mtDNA copy numbers are reduced to <200 per cell. This decrease in copy number, together with the random segregation of mtDNA molecules to daughter cells, can result in a markedly skewed distribution of mutation when mature oocytes are formed.^8, 10, 11^

Approximately 30 000 women per year are offered invasive prenatal testing in the United Kingdom.^12^ Included in this number is a small, but growing, group of women who are offered prenatal testing for mitochondrial disease. There is no cure for mitochondrial disease and effective treatments are lacking. Reproductive options for families affected by mitochondrial disease are important, particularly those who have already lost a child to the disease or for those women known to harbour mtDNA mutations. Prenatal testing for mitochondrial disease is only possible, however, with known causative mutations, where there is sufficient evidence from segregation and disease linkage studies, biochemical and functional assays to confirm the pathogenicity of these mutations. The primary aim of prenatal diagnosis for mitochondrial disease is to provide an accurate assessment of the risk of the foetus developing mitochondrial disease either *in utero* or in childhood.^13, 14, 15, 16^

Where mitochondrial disease is inherited in an autosomal recessive manner, as is most often the case in childhood-onset mitochondrial disease, and the genetic changes identified are novel, then carrier status should be confirmed in each parent with additional evidence provided from functional studies supporting pathogenicity. When a mtDNA mutation is responsible, then heteroplasmy levels in blood and urine should be determined in the mother and, where possible, in maternal relatives, especially previously affected children. For a minority of mtDNA mutations, there is a clear correlation between the level of heteroplasmy and disease severity, but this does differ between families and assessment of foetal risk should be made in the context of how other family members have been affected.^16^

Since establishing the nationally commissioned mitochondrial diagnostic service in 2007, we have experienced an increasing demand for prenatal diagnostic testing from families affected by mitochondrial disease. We have retrospectively reviewed the analyses performed over the period April 2007 to January 2013 to inform the debate and plan future testing strategies.

## Materials and Methods

Data regarding prenatal diagnostic testing were collected from two (Newcastle and Oxford) of the three centres (Newcastle, Oxford and London) commissioned by the National Health Service to provide a clinical and diagnostic service for Rare Mitochondrial Diseases of Adults and Children (the service in London did not undertake any prenatal investigations during this period). We are not aware of any other genetic testing centres in the United Kingdom undertaking mtDNA heteroplasmy assessment and although it is possible that other centres have performed prenatal assessment for nuclear mutations, the availability of a free nationally commissioned service makes this somewhat unlikely. Three reviewers examined the diagnostic reports and medical notes over the 69-month period between April 2007 and January 2013. Discrepancies and omissions in the clinical information were clarified with the clinical team.

### Assessing mutation status during diagnostic chorionic villus biopsy (CVB) testing

A variety of molecular techniques are used for prenatal testing depending upon the causative familial mutation. Presence or absence of nuclear gene mutations is ascertained by direct Sanger sequencing of PCR-amplified products. Prenatal assessment of heteroplasmy levels of familial mtDNA point mutations was achieved using mutation-specific pyrosequencing assays.^17^ These assays have been established in the Newcastle laboratory for a variety of pathogenic mutations and have been shown to be sensitive in their detection of 1% mutation load.^18, 19, 20, 21^ Mutation screening for large-scale single mtDNA deletions is performed using a combined methodology involving quantitative fluorescent real-time PCR (QPCR) and long-range PCR.^22^ The QPCR assay compares the copy number of the commonly deleted *MTND4* gene against the copy number of the *MTND1* gene, which is seldom deleted in patients with large-scale mtDNA rearrangements.^23^

## Results

Sixty-two prenatal diagnoses were made in the 69-month study period, of which 59 were CVB samples and 3 were amniocentesis. Gestation at time of testing ranged from 8 weeks 5 days to 15 weeks for CVB, with the amniocenteses being performed between 15 weeks 5 days and 17 weeks 3 days. Prenatal testing was requested in the majority of patients as a consequence of having had a previously affected child (*n*=58). Other requests were due to being a known mtDNA mutation carrier (although clinically asymptomatic; *n*=3), or having severely affected siblings (*n*=1).

### mtDNA mutations

Of the prenatal samples assessed, 17 were for mutations in mtDNA (m.3243A>G (*n*=4), m.11777C>A (*n*=2), m.9176T>C (*n*=2), m.14453G>A (*n*=1), m.13513G>A (*n*=1), m.8344A>G (*n*=1), m.8993T>G (*n*=1), m.8993T>C (*n*=1), m.10191T>C (*n*=1), m.10158T>C (*n*=1), m.3688G>A (*n*=1) and a single, large-scale mtDNA deletion (*n*=1); Table 1). In addition to accurately determining the level of mtDNA mutation in the foetal DNA sample, all mtDNA mutations were further categorised as having low (<30%), intermediate (30–70%) or high (>70%) mtDNA loads. Of the 17 prenatal samples, high heteroplasmy levels were detected in CVB samples from patients 6 and 8 and in both cases termination of pregnancy was performed. Patient 8 herself harboured relatively high levels of the m.8993T>G mutation in blood DNA (73% heteroplasmy) although the threshold for a severe neurological phenotype, such as Leigh Syndrome, in association with the m.8993T>G mutation is typically very high.^24^ In contrast, patient 6 did not have the mutation detectable in her blood or urine. Intermediate heteroplasmy levels were identified in six samples (patients 1, 5, 9, 12, 14 and 16), one of which resulted in termination of pregnancy (patient 1), and the outcome for the remainder is not known. Five of these mothers themselves had intermediate heteroplasmy levels of the respective mutation in DNA derived from their urinary epithelium; one had high heteroplasmy levels (patient 9), but (as is the case for the m.8993T>G and indeed the m.9176T>C mutation) the m.8993T>C mutation is associated with a very high threshold.^24, 25, 26^ Low level mutation load was detected in the CVB samples from patients 7 and 15, both of whom continued their pregnancies. Patient 15, who harboured low heteroplasmy levels in blood and intermediate levels in buccal and urinary epithelium of m.3688G>A, went on to deliver a clinically unaffected child. The outcome for the child born to patient 7, in whom we could not detect the familial m.9176T>C mutation in blood or urine, is not known. The familial mutation was not detected in seven prenatal samples, from four mothers in whom we found no evidence of the familial mutation in blood or urine (patient 2 (m.3243A>G), patient 4 (m.3243A>G), patient 10 (m.14453G>A), patient 17 (single deletion)), two mothers harbouring low heteroplasmy levels (patient 3 (m.3243A>G) and patient 13 (m.10191T>C)) and one mother with intermediate levels in blood (patient 11 (m.13513G>A)). Postnatal screening for the familial mutation was performed for three cases using blood samples; the results identified no evidence of the corresponding mutation in each instance and they remain clinically well (patient 4 (m.3243A>G), patient 10 (m.14453G>A) and patient 11 (m.13513G>A)).

### nDNA mutations

The remaining 45 prenatal samples were screened for recessive nDNA mutations (*POLG* (*n*=19), *SURF1* (*n*=14), *NDUFS2* (*n*=2), *MPV17* (*n*=2), *RARS2* (*n*=2), *DGUOK* (*n*=2), *NDUFV1* (*n*=1), *TK2* (*n*=1), *SUCLA2* (*n*=1), *TMEM70* (*n*=1)) (Supplementary Table 2). Of these, 12 inherited both parental mutations, 8 of which resulted in termination of the pregnancy, 2 pregnancies continued resulting in the birth of affected infants (mutations confirmed on infant blood DNA for one case) and the outcome of the remaining 2 cases is not known. There were 15 heterozygous carriers and the familial mutations were not detected in 18 cases. Pregnancy was terminated in one of these cases for an incidental finding of the XXY karyotype (patient 26).

## Discussion

The aim of prenatal diagnostic testing in mitochondrial disease is to identify foetuses harbouring mutations that will cause severe disease and offer termination at a relatively early stage of pregnancy. In our experience, prenatal testing is usually requested as a consequence of having had a previously affected child (57/62 requests).

CVB, usually performed at 11–14 weeks gestation, is the procedure most commonly offered to women at risk of having a child affected by mitochondrial disease, and particularly for those harbouring mtDNA mutations. Amniocentesis, a procedure that relies on aspirating cellular material (largely shed foetal epithelial cells) from the amniotic sac, may be less reliable in detecting and quantifying mtDNA mutations and tends to be offered to those women presenting later in pregnancy or where a nDNA mutation is responsible for the mitochondrial disease. Prenatal diagnostic testing procedures also bring additional risk of miscarriage; approximately 1% and 2% higher than spontaneous miscarriage for amniocentesis and CVB, respectively.^12^ No spontaneous miscarriages occurred as a result of prenatal testing in our cohort. Percutaneous umbilical blood sampling is not routinely offered in mitochondrial disease because of the risk of maternal contamination of the sample and the increased risk of foetal loss (<3%) associated with the procedure.^12^ We do not offer a biochemical prenatal diagnosis, although this has been performed (rarely) at other international centres where a biochemical deficiency has been established in a previously affected child, but no genetic diagnosis achieved. This type of biochemical diagnosis has a number of specific requirements and as a result worldwide experience is very limited.^16^

For women known to harbour pathogenic mtDNA mutations, interpretation of prenatal testing is complex and it is important to consider factors such as heteroplasmy of the mtDNA mutation, threshold levels, phenotypic expression of the mutation in maternal relatives and the strength of association between genotype and phenotype. Only through evaluating all of these aspects can the clinician provide clear, accurate genetic advice regarding the likely outcome for the foetus affected by a mtDNA mutation.^13, 15, 16, 20^ Recent studies indicate that prenatal samples provide an accurate prediction of mutation load in the postnatal period.^27^ In addition, studies analysing heteroplasmy levels in blastomeres from disaggregated eight cell stage embryos, have indicated that there is less variation between cells than was previously considered to be the case, implying that sampling bias in prenatal diagnosis is minimal.^28^ Further research to ascertain the correlation between genotype, mutation load and phenotype is required with regards to some mtDNA mutations. In the seven cases in which the mutation was not detected, these families could be reassured that their child would not be clinically affected by mitochondrial disease. Counselling mothers with foetuses harbouring mtDNA mutations in the intermediate range (*n*=11) can prove more difficult particularly for mutations where the relationship between genotype and phenotype is far less clear (eg, m.3243A>G). In this context, information regarding levels of heteroplasmy in affected and unaffected close relatives is very important. This was particularly true for patient 1 (Table 1), who was the only intermediate result to lead to termination of pregnancy. Extensive pre- and post-result counselling included a discussion of m.3243A>G heteroplasmy levels present in patient 1's affected and unaffected maternal relatives, who gave permission to disclose this information for the purposes of counselling. The two women with foetuses demonstrating high heteroplasmy levels on prenatal testing both opted for termination of pregnancy following appropriate counselling with both a specialist in mitochondrial disease and a genetic counsellor. Although all families affected by mitochondrial disease should be aware of available reproductive options, it is these women, harbouring high levels of pathogenic mtDNA mutations, who may wish to consider alternative options for future pregnancies. Such options include gamete (egg or sperm) donation, or adoption as an alternative to pregnancy. Techniques to reduce or prevent transmission of mtDNA and nDNA mutations, such as pre-implantation genetic diagnosis, are already available.^14, 29, 30^

One of the shortfalls of this retrospective collection of data on prenatal diagnoses in patients with mitochondrial disease is the paucity of information available regarding follow-up on children born after prenatal diagnosis. It appears that for mothers with low or intermediate levels of mtDNA mutation, routine outpatient follow-up is infrequent and therefore information regarding their children scant. However, as a national service, we are not aware of any children presenting with mitochondrial disease symptoms following prenatal mtDNA diagnosis during the period April 2007–January 2013.

Interpretation of prenatal testing is relatively straightforward in those families harbouring nDNA mutations where classic Mendelian rules of inheritance apply. Parents can be given much clearer results with regards as to whether or not their child will be affected. In keeping with current UK practice, at parental request we have also provided information on the carrier status of the foetus.^31^

For homoplasmic maternally transmitted mtDNA disease, where PGD is not an option, further strategies are being developed. Techniques such as pronuclear transfer, where male and female pronuclei from the patient's fertilised oocyte are transferred to an enucleated healthy fertilised donor oocyte, and metaphase II spindle transfer, which involves the transfer of the metaphase II spindle from a mature affected oocyte into an enucleated healthy donor oocyte before fertilisation, are at an advanced stage of development with successful metaphase II spindle transfer having been performed in Macaque monkeys.^32, 33, 34^ In March 2013, the Human Fertilisation and Embryology Authority (HFEA) agreed its advice to UK Government on the ethics and science of IVF-based techniques designed to prevent the transmission of maternally inherited mitochondrial disease. Further work assessing the safety, efficacy and applicability of this technique is planned (www.hfea.gov.uk/6896.html).

## Conclusion

We have experienced an increasing demand for prenatal diagnostic testing from families affected by mitochondrial disease, with 62 prenatal diagnoses made since the service was first offered in 2007 and at least 11 cases of mitochondrial disease prevented.

Information on reproductive options including prenatal diagnosis (CVB or amniocentesis), egg donation and sperm donation, techniques to reduce or prevent transmission (eg, PGD), or adoption as an alternative to pregnancy, should be presented to all families affected by mitochondrial disease to facilitate informed reproductive choices. Pronuclear transfer and metaphase II spindle transfer continue to be developed, but are not yet available. The present availability of PGD and the future possibility of nuclear transfer mark an exciting phase in preventing the transmission of mitochondrial disease.

## Figures and Tables

**Table 1:** Prenatal testing for mtDNA mutations

*Pt*	*Reason for CVB*	*Screening mutation*	*Maternal mutation and heteroplasmy level*	*Result of CVB*	*Clinical outcome*
1	Mother known mutation carrier	m.3243A>G	m.3243A>G (55% urine, 12% blood)	68% m.3243A>G^a^	Termination of pregnancy
2	Previously affected child	m.3243A>G	m.3243A>G not detected in blood/urine/buccal samples	No mutation detected	Pregnancy continued
3	Mother known mutation carrier	m.3243A>G	m.3243A>G (1% blood, 18% urine)	No mutation detected	Pregnancy continued
4	Maternal grandmother and brother affected	m.3243A>G	m.3243A>G not detected in blood/urine	No mutation detected	Baby clinically well; no mutation detected in blood DNA at 11 weeks
5	Previously affected child	m.8344A>G	m.8344A>G (35% blood, 35% urine)	46% m.8344A>G^a^	Data not available
6	Previously affected child	m.9176T>C	m.9176T>C not detected in blood/urine	98% m.9176T>C^a^	Termination of pregnancy
7	Previously affected child	m.9176T>C	m.9176T>C not detected in blood/urine	9% m.9176T>C^a^	Pregnancy continued
8	Previously affected child	m.8993T>G	m.8993T>G (73% blood)	98% m.8993T>G^a^	Termination of pregnancy; PM samples confirmed mutation levels 97%
9	Previously affected child	m.8993T>C	m.8993T>C (79% blood, 86% urine, 82% buccal)	58% m.8993T>C^a^	Pregnancy continued
10	Previously affected child	m.14453G>A	m.14453G>A not detected	No mutation detected	Clinically unaffected baby girl born
11	Previously affected child	m.13513A>G	m.13513G>A (45% blood)	No mutation detected	Baby clinically well; no mutation detected in blood DNA at 12 weeks
12	Maternal brother affected	m.11777C>A	m.11777C>A (30% blood, 36% urine, 43% buccal)	46% m.11777C>A^a^	Data not available
13	Mother known mutation carrier	m.10191T>C	m.10191T>C (2% blood, 16% urine)	No mutation detected	Pregnancy continued
14	Previously affected child	m.10158T>C	m.10158T>C (5% blood, 33% urine, 16% buccal)	52% m.10158T>C^a^	Data not available
15	Previously affected child	m.3688G>A	m.3688G>A (20% blood, 50% urine, 43% buccal)	3% m.3688G>A^a^	Clinically unaffected baby girl born
16	Previously affected child	m.11777C>A	m.11777C>A (18% blood, 32% urine, 20% buccal)	54% m.11777C>A^a^	Alive and well at 5 years
17	Previously affected child	Single large-scale mtDNA deletion	No deletion in mtDNA detected	mtDNA deletion not detected	Pregnancy continued

Seventeen women referred for prenatal testing because of a personal or maternal family history of mtDNA disease, results of the prenatal test and clinical outcome where known.

Reference sequence: Revised Cambridge Reference Sequence;^35^ GenBank Reference NC_012920.1.

a Information submitted to the publicly available MITOMAP database (http://www.mitomap.org/MITOMAP).
