# Supplementary material for: A national perspective on prenatal testing for mitochondrial disease
Source: Eur J Hum Genet. 2014 Mar 19;22(11):1255–9. doi: 10.1038/ejhg.2014.35 (PMC4200441; doi:10.1038/ejhg.2014.35)
Supplement: Supplementary Table 2 [file ejhg201435x1.doc]

**Supplementary Table 2**: Prenatal testing for nDNA mutations

| **Pt** | **Reason for CVB** | **Nuclear gene** | **Maternal mutation** | **Paternal mutation** | **Results of CVB** | **Clinical outcome** |
| --- | --- | --- | --- | --- | --- | --- |
| 18 | Previously affected child  (Alpers’ syndrome) | *POLG* | c.1399G>A p.(Ala467Thr) | c.1283T>C p.(Leu428Pro) | c.1399G>A carrier | Clinically unaffected |
| 19 | Previously affected child  (Alpers’ syndrome) | *POLG* | c.[695G>A;830A>T] p.([Arg232His; His277Leu]) | c.926G>T p.(Arg309Leu) | c.[695G>A;830A>T] carrier | Data not available |
| 20 | Previously affected child  (Alpers’ syndrome) | *POLG* | c.2542G>A p.(Gly848Ser) | c.1399G>A p.(Ala467Thr) | No mutation detected | Data not available |
| 21 | Previously affected child  (Alpers’ syndrome) | *POLG* | c.1399G>A p.(Ala467Thr) | c.2740A>C p.(Thr914Pro) | Affected, c.[1399G>A];[2740A>C] | Termination of pregnancy |
| 22 | Previously affected child  (Alpers’ syndrome) | *POLG* | c.2740A>C p.(Thr914Pro) | c.1399G>A p.(Ala467Thr) | No mutation detected | Data not available |
| 23 | Previously affected child  (Alpers’ syndrome) | *POLG* | c.2740A>C p.(Thr914Pro) | c.2243G>C p.(Trp748Ser) | No mutation detected | Data not available |
| 24 | Previously affected child  (Alpers’ syndrome) | *POLG* | c.2740A>C p.(Thr914Pro) | c.2243G>C p.(Trp748Ser) | c.2243G>C carrier | Baby clinically unaffected |
| 25 | Previously affected child  (Alpers’ syndrome) | *POLG* | c.2554C>T p.(Arg852Cys) | c.1399G>A p.(Ala467Thr) | No mutation detected |  |
| 26 | Previously affected child  (Alpers’ syndrome) | *POLG* | c.1879C>T p.(Arg627Trp) | c.2740A>C p.(Thr914Pro) | No mutation detected | Termination of pregnancy as 47XXX identified |
| 27 | Previously affected child  (Alpers’ syndrome) | *POLG* | c.2897T>G p.(Leu966Arg) | c.1399G>A p.(Ala467Thr) | c.1399G>A carrier | Data not available |
| 28 | Previously affected child  (Alpers’ syndrome) | *POLG* | c.1399G>A p.(Ala467Thr) | c.2542G>A p.(Gly848Ser) | Affected, c.[1399G>A];[2542G>A] | Pregnancy continued; clinically affected boy born, mutation status confirmed on blood DNA |
| 29 | Previously affected child  (Alpers’ syndrome) | *POLG* | c.2542G>A p.(Gly848Ser) | c.1399G>A p.(Ala467Thr) | Affected, c.[1399G>A];[2542G>A] | Termination of pregnancy |
| 30 | Previously affected child  (Alpers’ syndrome) | *POLG* | c.2542G>A p.(Gly848Ser) | c.1399G>A p.(Ala467Thr) | Affected, c.[1399G>A];[2542G>A] | Termination of pregnancy |
| 31 | Previously affected child  (Alpers’ syndrome) | *POLG* | c.2740A>C p.(Thr914Pro) | c.1399G>A p.(Ala467Thr) | Affected, c.[1399G>A];[2740A>C] | Termination of pregnancy |
| 32 | Previously affected child  (Alpers’ syndrome) | *POLG* | c.2740A>C p.(Thr914Pro) | c.1399G>A p.(Ala467Thr) | c.1399G>A carrier | Ongoing pregnancy |
| 33 | Previously affected child  (Alpers’ syndrome) | *POLG* | c.1399G>A p.(Ala467Thr) | c.2246T>C p.(Phe749Ser) | c.2246T>C carrier | Data not available |
| 34 | Previously affected child  (Alpers’ syndrome) | *POLG* | c.[1760C>T;1765C>A] p.([Pro587Leu;Pro589Thr]) | c.2542G>A p.(Gly848Ser) | Affected, c.[1760C>T;1765C>A];[2542G>A] | Data not available |
| 35 | Previously affected child  (Alpers’ syndrome) | *POLG* | c.2542G>A p.(Gly848Ser) | c.1399G>A p.(Ala467Thr) | Affected, c.[1399G>A];[2542G>A] | Termination of pregnancy |
| 36 | Previously affected child  (Alpers’ syndrome) | *POLG* | c.2542G>A p.(Gly848Ser) | c.1399G>A p.(Ala467Thr) | Affected, c.[1399G>A];[2542G>A] | Termination of pregnancy |
| 37 | Previously affected child | *SURF1* | c.799_800delCT p.(Leu267Glufs*24) | c.799_800delCT p.(Leu267Glufs*24) | c.799_800delCT carrier | Data not available |
| 38 | Previously affected child | *SURF1* | c.799_800delCT p.(Leu267Glufs*24) | c.799_800delCT p.(Leu267Glufs*24) | Affected, homozygous c.799_800delCT | Data not available |
| 39 | Previously affected child | *SURF1* | c.324-11T>G p.(Asp108_Gly172delinsGlu) splice mutation | c.324-11T>G p.(Asp108_Gly172delinsGlu) splice mutation | c.324-11T>G carrier | Data not available |
| 40 | Previously affected child | *SURF1* | c.799_800delCT p.(Leu267Glufs*24) | c.799_800delCT p.(Leu267Glufs*24) | c.799_800delCT carrier | Data not available |
| 41 | Previously affected child | *SURF1* | c.799_800delCT p.(Leu267Glufs*24) | c.799_800delCT p.(Leu267Glufs*24) | No mutation detected | Data not available |
| 42 | Previously affected child | *SURF1* | c.799_800delCT p.(Leu267Glufs*24) | c.799_800delCT p.(Leu267Glufs*24) | No mutation detected | Data not available |
| 43 | Previously affected child | *SURF1* | c.799_800delCT p.(Leu267Glufs*24) | c.799_800delCT p.(Leu267Glufs*24) | No mutation detected | Data not available |
| 44 | Previously affected child | *SURF1* | c.312_321delinsAT p.(Leu105*) | c.312_321delinsAT p.(Leu105*) | No mutation detected | Data not available |
| 45 | Previously affected child | *SURF1* | c.792_793delAG p.(Arg264Serfs*27) | c.792_793delAG p.(Arg264Serfs*27) | No mutation detected | Data not available |
| 46 | Previously affected child | *SURF1* | c.799_800delCT p.(Leu267Glufs*24) | c.799_800delCT p.(Leu267Glufs*24) | No mutation detected | Data not available |
| 47 | Previously affected child | *SURF1* | c.833+1G>A p.(Gln251Argfs*67) splice mutation | c.833+1G>A p.(Gln251Argfs*67) splice mutation | c.833+1G>A carrier | Data not available |
| 48 | Previously affected child | *SURF1* | c.312_321delinsAT p.(Leu105*) | c.312_321delinsAT p.(Leu105*) | c.312_321delinsAT carrier | Data not available |
| 49 | Previously affected child | *SURF1* | c.799_800delCT p.(Leu267Glufs*24) | c.799_800delCT p.(Leu267Glufs*24) | c.799_800delCT carrier | Data not available |
| 50 | Previously affected child | *SURF1* | c.312_321delinsAT p.(Leu105*) | c.312_321delinsAT p.(Leu105*) | No mutation detected | Data not available |
| 51 | Previously affected child | *RARS2* | c.1211T>A p.(Met404Lys) | c.472_474delAAA p.(Lys158del) | No mutation detected | Data not available |
| 52 | Previously affected child | *RARS2* | c.1024A>G p.(Met342Val) | c.35A>G p.(Gln12Arg) | No mutation detected | Data not available |
| 53 | Previously affected child  (Leigh syndrome) | *NDUFS2* | c.353G>A p.(Arg118Gln) | c.875T>C p.(Met292Thr) | No mutation detected | Baby clinically unaffected |
| 54 | Previously affected child  (Leigh syndrome) | *NDUFS2* | c.413G>A p.(Arg138Gln) | c.998G>A p.(Arg333Gln) | No mutation detected | Pregnancy ongoing |
| 55 | Previously affected child | *NDUFV1* | c.929G>T p.(Gly310Val) | c.1080G>A splice mutation | Affected, c.[929G>T];[1080G>A] | Termination of pregnancy |
| 56 | Previously affected child^  (hepatocerebral depletion) | *MPV17* | c.71-?_(*420_?)del (exons 3-8 deletion) | c.71-?_(*420_?)del (exons 3-8 deletion) | c.71-?_(*420_?)del carrier | Data not available |
| 57 | Previously affected child (hepatocerebral depletion) | *MPV17* | c.71-?_(*420_?)del (exons 3-8 deletion) | c.71-?_(*420_?)del (exons 3-8 deletion) | Affected, homozygous  c.71-?_(*420_?)del | Affected female born |
| 58 | Previously affected child^ (hepatocerebral depletion) | *DGUOK* | c.115C>T p.(Arg39*) | c.115C>T p.(Arg39*) | No mutation detected | Pregnancy continued |
| 59 | Previously affected child (hepatocerebral depletion) | *DGUOK* | c.679G>A p.(Glu227Lys) | c.591G>A splice mutation | c.591G>A carrier | Data not available |
| 60 | Previously affected child  (mtDNA depletion myopathy) | *TK2* | c.173A>G p.(Asn58Ser) | c.133C>T p.(Gln45*) | Affected, c.[133C>T];[173A>G] | Termination of pregnancy |
| 61 | Previously affected child | *SUCLA2* | c.1271delG p.(Gly424Aspfs*18) | c.1271delG p.(Gly424Aspfs*18) | No mutation detected | Clinically unaffected baby girl born |
| 62 | Previously affected child | *TMEM70* | c.317-2A>G splice mutation | c.317-2A>G splice mutation | c.317-2A>G carrier | Pregnancy continued |

***Table 2: 50 women referred for prenatal testing due to a history of nDNA disease, results of the prenatal test and clinical outcome where known***

***KEY: ^ Amniocentesis N/A - not applicable***

***GenBank Reference Sequences:***

***POLG – NM_002693.2; SURF1 – NM_003172.2; RARS2 – NM_020320.3; NDUFS2 – NM_004550.4; NDUFV1 – NM_007103.3; MPV17 – NM_002437.4; DGUOK – NM_080916.1; TK2 – NM_004614.4; SUCLA2 – NM_003850.2; TMEM70 – NM_017866.5***

Mutation information submitted to the Human DNA Polymerase Gamma Database (<http://tools.niehs.nih.gov/polg/>) and the Leiden Open Variation Database (LOVD) 3.0 Shared Installation (<http://www.lovd.nl/GENESYMBOL>)
